# Zinc-Induced Copper Deficiency as a Rare Cause of Neurological Deficit and Anemia

**DOI:** 10.7759/cureus.43856

**Published:** 2023-08-21

**Authors:** Nithin Gupta, Martin F Carmichael

**Affiliations:** 1 Medicine, Campbell University School of Osteopathic Medicine, Lillington, USA; 2 Family Medicine, Conway Medical Center, Conway, USA

**Keywords:** hypocupremia, neurologic defecit, anemia, zinc, copper

## Abstract

Upper respiratory infections (URIs) are common and carry a large economic burden due to missed work and school. This has prompted an increased interest in over-the-counter zinc supplementation to enhance immunity and reduce illness duration. Zinc's antiviral and anti-inflammatory effects have led to its inclusion in popular URI medications and a surge in supplement sales, particularly among the elderly. However, zinc over-supplementation in this population can lead to hypocupremia, causing various presentations such as anemia, paresthesia, and gait disturbances. Here, we present a case of a 76-year-old female who developed hypocupremia due to zinc supplementation. Her initial presentation involved an unsteady gait, and severe anemia was detected during the examination. The patient's condition required hospital admission, and subsequent investigations confirmed severe pancytopenia and low blood copper levels. Discontinuation of zinc supplementation and oral copper gluconate administration led to a full recovery of her anemia and cell count; however, her neurological deficits remain. This case highlights the importance of counseling patients on the potential adverse effects of zinc supplementation and brings to light a potentially overlooked diagnosis, particularly in the elderly population.

## Introduction

Upper respiratory infections (URIs) are one of the most common and burdensome diagnoses in the United States, accounting for annual costs exceeding $22 billion [1]. With the recent COVID-19 pandemic, there has been greater interest in over-the-counter (OTC) zinc supplementation, aimed at boosting immunity and decreasing the length of illness [2]. There are multiple studies that report the antiviral effects of zinc on viruses commonly implicated in URIs (such as influenza and rhinovirus), anti-inflammatory effects in the lung, and improved leukocyte recruitment/function [2-4]. These findings have led to the inclusion of zinc in popular OTC cold medications as well as increased sales of zinc supplements, with the global market expected to double by 2030 [5]. In the United States, a large majority of users of zinc supplementation are those 71 years and older, who favor it due to the suggested anti-inflammatory/oxidant effects and prevention of neurodegeneration [6]. Although there are noted benefits to supplementation, there are a relatively small number of reports that describe zinc over supplementation in the elderly leading to hypocupremia [7]. These individuals may have variable presentation, which may include, anemia, dizziness, paresthesia, and/or gait disturbances [8]. In this report, we seek to add to the growing body of literature by presenting the case of an elderly female who developed hypocupremia due to zinc supplementation.

## Case presentation

Initial presentation

A 76-year-old Caucasian female presented to our clinic in 2019 with a chief complaint of unsteady gait and described “drifting to the right” over the last several weeks. The patient’s past medical history included hyperlipidemia and hypothyroidism, both well-controlled. In addition to prescription medication for the aforementioned diseases, the patient was also taking aspirin (81 mg) and various OTC daily supplements, including chondroitin-glucosamine, ICaps AREDS, magnesium oxide (400 mg), CoQ10 (300 mg), and zinc (50 mg).

On exam, the patient was afebrile and stable, with a blood pressure of 170/78 mmHg and SpO2 of 96%. Physical examination revealed an unremarkable nose and throat, no nystagmus with position change, no dysphagia or speech deficit, and no muscle weakness or numbness. The patient demonstrated drifting of gait to the right while walking. Upon blood examination for complete blood count and basic metabolic panel, it was noted that the patient was severely anemic. The patient’s hemoglobin was measured to be 6 g/dL (reference: 12.0-16.0 g/dL, fingerstick measurement) with no signs of rectal or GI bleeding. The patient was then sent to the emergency room.

Hospital course

The patient was admitted to the hospital where further lab work was done prior to the infusion of one unit of packed red blood cells (pRBCs). A stroke workup, brain MRI, and head/neck CT angiography (CTA) were performed. MRI of the cervical spine with and without contrast was performed, which demonstrated possible abnormal cord signal posteriorly at the level of C2 through C4-5 (however, motion artifact could not be ruled out). The next day, the patient’s blood work showed severe pancytopenia and normal folic acid (low end of normal range), which was being replaced orally. Further, serum iron, total iron binding capacity (TIBC), and vitamin B12 levels were all within normal range. Hemolytic anemia was also ruled out and further labs showed leukopenia and a normal reticulocyte count. Hematology was consulted and a bone marrow biopsy demonstrated findings suggestive of myelodysplastic syndrome (vacuolization of erythroid/myeloid precursor cells, hypercellularity of bone marrow, and atypical cell morphology). Due to these abnormalities, an IntelliGEN panel (to evaluate myeloid chromosomal mutations) and serum protein electrophoresis were performed, which showed no significant clinical variant and an unobserved M-spike, respectively. Finally, a copper level was taken and the results showed a copper level < 0.50 ug/mL (reference: 0.50-1.50). The patient was informed that the zinc supplement she had been ingesting daily was potentially the cause of the copper deficiency, and it was recommended that she discontinue use. She stated that she had previously had a URI 11 months prior and had heard zinc supplementation may shorten the course of the infection. She started taking zinc 11 months prior during her URI and had continued supplementation since then. Prior to discharge, the patient received one more unit of pRBCs (due to low hemoglobin) and was started on copper gluconate 2 mg, twice daily.

Outcome

The patient was seen two months after the initial diagnosis, whereupon a complete blood count demonstrated improved counts in all cell lines and increased hemoglobin and hematocrit. The patient has also exhibited recovery in her previously impaired gait and neurological deficits, although some slight deficiencies remain. Her copper level three months after supplementation started was 0.79 (reference: 0.50-1.50). Her hemoglobin levels have been within normal range and her most recent blood work in 2023 showed all values within the normal reference range (Table 1). The temporal trends in the patient’s hemoglobin level, copper level, and cell count are further summarized in Table 1.

**Table 1:** Summary of pertinent lab findings at the time of diagnosis, two months after zinc cessation and addition of oral copper gluconate, and most recent levels.

	At the time of diagnosis	Two months post-treatment	Most recent	Reference range
WBC	2.1	4.2	5.7	4.5-11.0 (K/mm^3)
RBC	2.25	3.57	3.92	4.00-5.00 (1326M/mm^3)
Hemoglobin	7.1	11.3	12.7	12.0-16.0 (g/dL)
Hematocrit	22.0	33.2	37.7	36.0-46.0 (%)
Absolute neutrophil	0.6	2.6	3.8	1.8-7.7 (K/mm^3)
Copper	<0.50	0.79	1.26	0.50-1.50 ug/mL

## Discussion

Zinc-induced hypocupremia, although relatively uncommon, is an important cause of anemia and/or neurological deficit in the elderly. When excessive zinc is present in the body, there is increased production of the binding protein metallothionein, which helps in copper absorption [8]. The increased metallothionein produced by enterocytes causes copper to be trapped in the enterocytes and lost in the feces when the intestinal epithelium is lost and excreted in the feces [9].

The complex interactions of copper and iron, which lead to anemia, have not been fully elucidated. It has been suggested that copper is critical in the differentiation of hematopoietic stem cells, and low copper levels cause mitochondrial reprogramming, which leads to an expansion state (rather than differentiating into progenitors) [10]. In addition, copper also plays a role in intestinal iron absorption through the alteration of DNA-binding proteins, all of which are factors that may account for the severe anemia seen in patients [11].

Although not reported in all cases of zinc-induced hypocupremia, neurological deficits, such as those seen in our patient, are not uncommon. Plantone et al. presented two cases of copper deficiency myelopathy, in which MRI demonstrated lesions of the posterior column of C2-3. Further, both patients had gait disturbances and paresthesia, similar to the findings of our patient [12]. It is well known that copper is critical to the synthesis and stabilization of myelin, and also plays a major role in multiple enzymatic pathways needed for proper function of the nervous system [13,14].

Currently, similar causes of zinc-induced hypocupremia have also been seen in patients using zinc-containing creams for acne treatment, dermatitis enteropathica, and wound creams [15]. Further, there is a multitude of studies that report zinc-containing denture cream as a cause, which may be particularly concerning especially in the elderly population [8,15]. Although the inciting event leading to zinc-induced hypocupremia may differ, the literature suggests that most patients have either normocytic or macrocytic anemia (often in addition to neutropenia and/or leukopenia) with or without neurological deficit [6,8,12].

In our patient, cessation of zinc supplementation and treatment with oral copper gluconate was sufficient to improve the patient’s anemia and pancytopenia over several months. This is similar to treatment reported in previous studies, although it has been reported that IV copper gluconate can be used when intestinal absorption is compromised [8]. These treatments generally are enough to recover the deficient cell lines and improve physical symptoms. Similarly, treatment using oral copper gluconate in our patient resulted in a complete recovery of hemoglobin levels and cell lines. Her neurological deficits and gait disturbances have also improved; however, she still has permanent difficulties with gait and muscle weakness.

## Conclusions

We present the case of an elderly female who presented to our clinic with an unsteady gait and paresthesia of the hands. After hospitalization for severe anemia, she was found to have zinc-induced hypocupremia, which resolved with copper supplementation. This report provides evidence for the consideration of zinc-induced hypocupremia as a potential cause of neurological deficit and pancytopenia in the elderly after more common causes, such as malignancy, have been ruled out. Thus, it is important for providers to be cognizant of the adverse effects of zinc supplementation and consider it a cause in instances such as the one presented in this case, after ruling out more common causes such as gastrointestinal bleeding or stroke.
